# Genome and transcriptome-wide association studies identify multiple novel loci for dementia with grain in Japanese

**DOI:** 10.1038/s10038-025-01438-7

**Published:** 2025-12-17

**Authors:** Risa Mitsumori, Kouichi Ozaki, Yuko Saito, Daichi Shigemizu, Atsushi Iwata, Shigeo Murayama, Masahiro Akishita, Tomio Arai, Shumpei Niida, Kenji Toba

**Affiliations:** 1 Medical Genome Center, Research Institute, National Center for Geriatrics and Gerontology, Obu, Aichi Japan; 2 RIKEN Center for Integrative Medical Sciences, Yokohama, Kanagawa Japan; 3 Department of Aging Research, Nagoya University Graduate School of Medicine, Nagoya, Aichi Japan; 4 Department of Cardiovascular Medicine, Hiroshima University Graduate School of Biomedical and Health Sciences, Hiroshima, Japan; 5 Department of Neuropathology (Bioresource Center, Brain Bank for Aging Research), Tokyo Metropolitan Institute for Geriatrics and Gerontology, Tokyo, Japan; 6 Department of Neurology, Tokyo Metropolitan Institute for Geriatrics and Gerontology, Tokyo, Japan; 7 Tokyo Metropolitan Institute for Geriatrics and Gerontology, Tokyo, Japan; 8 Department of Pathology, Tokyo Metropolitan Institute for Geriatrics and Gerontology, Tokyo, Japan; 9 Research Institute, National Center for Geriatrics and Gerontology, Obu, Aichi Japan

**Keywords:** Genome-wide association studies, Dementia

## Abstract

Argyrophilic grain (AG) is a common neurodegenerative accumulation of 4 repeat tau in dendritic spine. Dementia with grain (DG) is defined as AGs with a sole pathological basis for cognitive decline. As with other multifactorial diseases, DG could result from interactions of environmental and genetic factors. However, the genetic basis of DG is largely unknown. To clarify the genetic architecture of DG pathogenesis, we conducted a genome-wide association study (GWAS) with 214 DG cases versus 12,405 controls. We have identified a candidate locus associated with the risk of DG, the *SVIL* locus on chromosome 10, with genome-wide significance (rs11595141, *P* = 4.86 $\times$ 10^–8^) in the GWAS. Transcriptome-wide association analysis using summary statistics for DG-GWAS identified *DAPK2* (*P*_TWAS_ = 3.68 $\times$ 10^–5^) as a novel candidate causal gene for DG pathogenesis in the brain frontal cortex. The genetic association analysis for the *APOE* locus revealed that the *APOE* allele did not affect DG pathogenesis. We also identified new variants in the *MAPT* encoding tau protein that could potentially affect DG pathology. This is the first GWAS for DG, and our genetic findings provide biological and clinical insights into the pathogenesis of DG.

## Introduction

Argyrophilic grain (AG) is an age-associated neurodegenerative accumulation of four repeat (R) tau in dendritic spine, characterized by spindle- or comma-shaped structure detected by Gallyas silver stain [1] and anti-4 repeat tau isomorphic specific antibody (RD4) [2]. AGs started to accumulate in the ambient gyrus (Saito Stage 1), spread to the medial temporal lobe (Stage II), and extended to the frontal lobe (Stage III), causing cognitive decline. Dementia with grain (DG) is defined as AGs as a sole morphological basis for cognitive decline [3]. The diagnosis of DG was based on Gallyas stain and RD4 immunostain, as well as western blot and immune-electron microscopy, confirmed by cryo-electron microscopy (cryo-EM). In contrast, Alzheimer’s disease (AD) related pathologies, neurofibrillary tangles (NFTs) and neuropil threads were cofilament of 3 R and 4 R tau [4] and distinct from AGs.

Corticobasal degeneration (CBD) and progressive supranuclear palsy (PSP) are other neurodegenerative disorders with abnormal aggregates of 4 R tau [5, 6]. The CBD pathology presents tau inclusions in neurons and glia with tau astrocytic plaques, and extensive thread-like pathology in both gray and white matter [6]. PSP is a tauopathy with abnormal accumulation of tau protein within neurons as neurofibrillary tangles (NFTs), primarily in the basal ganglia, diencephalon, and brainstem with neuronal loss in globus pallidus, subthalamic nucleus, and substantia nigra. Abnormal tau also accumulates within oligodendroglia and astrocytes [5]. Both CBD and PSP are sporadic disorders, with few reports of familial cases [7, 8]. Genetic association studies have identified the *H1* haplotype of *MAPT* locus at chromosome 17q21 as a major genetic risk factor for CBD and PSP [9]. However, almost the entire Japanese population only has the *H1* haplotype [10]. Furthermore, recent GWAS with Caucasian subjects have identified the *MOBP* locus as the common risk factor for CBD and PSP [11, 12]. These data suggest overlaps in genetic architecture across different tauopathies. On the other hand, the genetic basis for DG is so far not well understood.

In this study, we aimed to identify the genetic components of DG using GWAS in the Japanese population. We conducted the expression quantitative trait locus (eQTL) analysis and transcriptome-wide association analysis (TWAS) and investigated the possible mechanisms of DG pathogenesis by functional annotation using GWAS data. In addition, we compared the frequencies of genetic variants for *APOE*, the strong genetic risk factor for AD, and *MAPT*, which encodes the protein tau, between DG, AD, and cognitively normal (CN) subjects.

## Materials and methods

### Subjects and DNA samples

All 16,634 genomic DNA samples and the corresponding clinical data were recruited from the National Center for Geriatrics and Gerontology (NCGG) Biobank and Brain Bank for Aging Research, Tokyo Metropolitan Institute for Geriatrics and Gerontology (TMIG), Japan. This included 214 samples from patients with autopsy-confirmed DG, 12,405 control samples from cognitively normal (CN) subjects and non-carrier control subjects (12,307 CN and 98 non-AGs, respectively), and 4015 samples from patients with AD (included possible- and proxy-AD) and mild cognitive impairment (MCI), all of whom were Japanese. 214 DG cases and 98 non-AGs from TMIG were performed to detect AGs by autopsy brains using the Gallyas-Braak method and RD4 immunohistochemistry. The distribution of AGs followed a stereotypic regional pattern and could be classified into Saito’s stages [3]. DG cases were categorized above Stage 2. Non-AG subjects were categorized as Stage 0: No grains are detected. We referred to the age at the time of initial diagnosis for NCGG samples and at the time of death because of autopsy for TMIG samples. Among DG patients, the median age was 83.7 years (67-104 years), and 46.2% were female. The CN subjects from NCGG Biobank had subjective cognitive complaints but normal cognition on the neuropsychological assessment with a comprehensive neuropsychological test, and Mini-Mental State Examination score >23 (average: 27.8 ± 1.9). Among control subjects, the average age was 83.7 years (24-96 years), and 54.8% were female. The patients with AD were diagnosed with probable or possible AD and MCI using the criteria of the National Institute on Aging Alzheimer’s Association workgroups [13, 14]. Among AD patients, the average age was 78.1 years (43-98 years), and 61.7% were female. Genomic DNA from NCGG biobank was extracted from peripheral blood leukocytes by standard protocols using a Maxwell RSC Instrument and a Maxwell RSC Buffy Coat DNA Kit (Promega, Madison, WI, USA). Genomic DNA from TMIG was extracted from the renal cortex using a standard phenol-chloroform procedure and kept at -80°C until use. All subjects provided written informed consent. This study was approved by the ethics committee of the NCGG and TMIG and conducted in accordance with the Declaration of Helsinki.

### Genotyping and quality control for GWAS

Genome-wide genotyping data for the 12,405 CN were downloaded from NCGG biobank. Genome-wide genotyping of all subjects was performed using the Infinium Asian Screening Array (Illumina, San Diego, CA, USA). We created a reference panel for imputation with high accuracy using the 1000 Genomes Project Phase 3 (1KGP 3 [May 2013 n = 2504]), and 3181 Japanese whole-genome sequence data from NCGG. We performed SNP imputation with minimac4 using the Japanese reference panel above. We used variants with an INFO score $\ge \,$0.7 in the association analysis. We first applied quality control (QC) filters to the subjects using PLINK 1.9[15]:(1) sex inconsistencies (--check-sex), (2) kinship coefficient (--genome 0.25), (3) genotype missingness (--mind 0.05), and (4) exclusion of outliers from the clusters of East Asian populations in a principal component analysis that was conducted together with 1000 Genomes Phase 3 data. We next applied QC filters to the variants: (1) genotyping efficiency or call rate (--geno 0.02), (2) minor allele frequency (MAF) (--maf 0.01), (3) Hardy–Weinberg equilibrium (--hwe 0.001), and (4) Strand orientation (--snps-only).

### GWAS

Logistic regression analysis adjusted for sex and age was performed using PLINK 1.9 (--logistic, --covar) [15]. Heritability, genetic correlation, and linkage disequilibrium (LD)-score regression were evaluated using LDSC (v1.0.1) [16–18]. All variants were annotated using ANNOVER (avsnp150) [19]. Regional association plots were generated using LocusZoom (http://locuszoom.org). To check for the secondary association signals, we conducted conditional analyses for the loci of interest by performing logistic regression on each lead variant. To check the consistency of imputed genotypes for the lead variants of interest, we used multiplex PCR-invader assay (Third Wave Technologies, Madison, WI, USA) [20] by using a QuantStudio 7 Flex Real-Time PCR System (Thermo Fisher Scientific, Waltham, MA, USA) and sequencing. The genomic inflation factor lambda and LD score regression intercept were computed with LDSC v1.0.1 software using the ‘baselineLD’, in which LD scores built from the 1000 Genomes phase 3. The analysis was restricted to Hap Map3 variants and excluded multiallelic variants and variants without an rsID, and also excluded variants in MHC region on chromosome 6.

### Genotyping accuracy

We evaluated the genotyping accuracy for the imputed SNPs using the following methods. For DG-GWAS, the accuracy of lead SNPs was validated by the PCR-invader assay [20].

### Genetic heritability

To estimate SNP heritability in DG subjects, we performed LD score regression [17, 18]. We used LD scores from East Asian populations in the 1000 Genomes dataset suitable for general LD score analyses (LD score regression intercept, heritability) [17].

### Genetic correlation

To analyze the genetic correlation between DG-GWAS and Japanese GWAS for different phenotypes, we used LDSC [16, 17]. We obtained summary statistics of Japanese GWAS data from the BioBank Japan (BBJ) database (PheWeb.jp) [21, 22]. Since PheWeb.jp (https://pheweb.jp) provides more than 220 phenotype summary statistics, we could not download all of them due to limited capacity. As DG is related to neurodegeneration, we first selected brain-related diseases (brain tumor, cerebral aneurysm, intracerebral hemorrhage, and ischemic stroke). We then included disorders (depression, epilepsy, Hashimoto’s disease, pulse pressure, myocardial infarction, type 1 diabetes, type 2 diabetes, and AID disease) that are associated with increased risk of dementia and memory impairment. Phenotypes such as BMI, gastric cancer, nephrotic syndrome, and systemic lupus erythematosus were excluded as they are not directly related to dementia, but rather to sarcopenia, which itself is linked to dementia. Therefore, sarcopenia-related phenotypes were also included. All GWAS summary statistics data were formatted for LD score regression filtering to HapMap3 SNPs. We used LD score files for East Asians produced by LDSC.

### Transcriptome-wide association study (TWAS)

To estimate the association between predicted gene expression levels and GWAS summary statistics, we conducted a TWAS using Functional Summary-based Imputation (FUSION) [23]. We used precomputed prediction models of gene expression in brain tissues (amygdala, anterior, caudate, cerebellar, cerebellum, cortex, frontal, hippocampus, hypothalamus, nucleus, putamen, spinal cord, and substantia) with the expression weight data in the GTEx v8 expression model from FUSION site (http://gusevlab.org/projects/fusion/), LD score files for East Asian produced by LDSC, and the summary statistics of DG-GWAS. We set the Bonferroni significance level, taking into account the number of genes available for each tissue.

### Association analysis for *MAPT* and *APOE* loci

Genotyping data in the *MAPT* (chr17:42,971,748-45,105,700) and *APOE* (chr19:44,409,011-46,412,650) loci for 4,015 AD subjects were downloaded from NCGG biobank. We performed logistic regression analysis on the *MAPT* and *APOE* loci to compare DG versus controls as well as DG versus AD, adjusting for age and sex using PLINK 1.9 (--logistic, --covar). Regional association plots were generated using LocusZoom (http://locuszoom.org).

## Results

### DG-GWAS

**Fig. 1:**
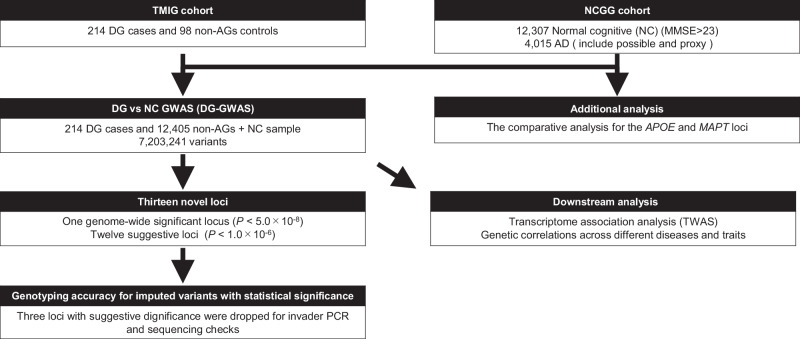
The workflow of this study

**Fig. 2:**
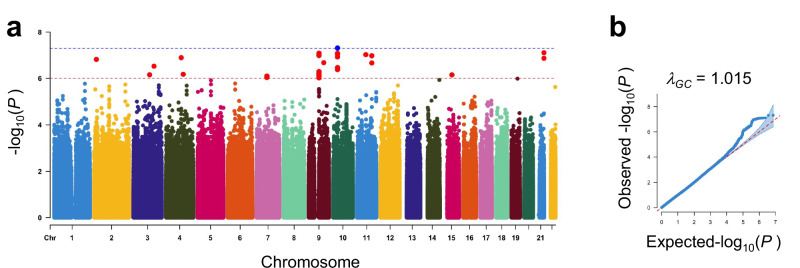
GWAS for DG. **a** A Manhattan plot of the DG-GWAS. **b** Quantile–quantile plot of the DG-GWAS *P*values. The blue line shows the GWAS significance threshold (*P* = 5.0$\times$10^–8^). The red line shows the suggestive threshold (*P* = 1.0$\times$10^–6^)

We illustrated the workflow of this study in Fig. 1. We conducted a DG-GWAS (214 DG cases and 12,405 control subjects, Supplementary Table S1) from the NCGG and TMIG biobanks. We selected control subjects with normal cognitive (MMSE > 23) from NCGG biobank, because DG was progressive neuronal loss with dementia [24]. We used 7,203,241 variants in the autosomes that passed QC filters. The genomic inflation factor (λ_GC_) was 1.02; the LD-score regression indicated that the inflation was primarily due to polygenic effects (LD-score regression intercept = 1.01). The estimated SNP heritability (*h*^*2*^) for observed score was 3.4% (standard error of the mean = 3.5%). The DG-GWAS identified a genome-wide significance (GWS) locus (*P* < 5.0$\times$10^–8^) and twelve suggestive loci (*P* < 1.0$\times$10^–6^) (Fig. 2a, b, and Supplementary Table S2). Among them, the three lead variants (rs147403806, rs140769784, and rs72732628) were directly determined by ASA genotyping. As the genotypes for the ten other lead variants were determined by imputation analysis, we examined the accuracy of the imputed genotyping data for the variants by using the PCR-invader assay and a subset of the DNA samples used in GWAS and evaluated the concordance rate (Number of mismatching genotypes/Number of subjects) of the imputed variants (Supplementary Table S3). The INFO scores for three variants exhibited above 0.7 (rs527654945, 0.89; rs78182510, 0.77; and rs141081800, 0.95); however, they showed low concordance (rs527654945, 94.57%; rs78182510, 94.58%; and rs141081800, 98.22%), and we excluded these from further analysis.

A GWS locus with lead SNP rs11595141 (*P* = 4.86$\times$10^–8^) was located closest to the *SVIL* gene (Table 1, Supplementary Fig. S1h). The nine remaining loci were *NBAS*, *SLC9A9*, *TET2*, *SYNPO2*, *CALN1*, *PGM5*, *PLPPR1*, *WDR72*, and *FAM207A* regions (Table 1, Supplementary Fig. S1a–g, i, j). All loci have no reports for genetic associations of dementia and related disorders (GWAS Catalog; https://www.ebi.ac.uk/gwas/). To verify the secondary association signals at these three DG-associated loci, we used the lead variants with 10 candidate loci in conditional analyses. No secondary independent association signals were detected in any of these loci (Supplementary Fig. S3).

**Table 1:** Summary statistics of genetic associations for the variants identified in DG-GWAS

Chr	dbSNP ID	Position (hg19)	A1	A2	Logistic regression											Annotated gene†	Location
					OR	95% CI	*P*	A1 allele frequency									
								DG	CN	All	gnomAD (v4.1.0)						
											All	European (Finnish)	African/African American	Admixed American	East Asian		
2	rs187486988	15340672	G	A	2.96	1.98–4.45	1.52E–07	0.072	0.028	0.029	1.51E–04	0	0	0	4.44E–03	*NBAS*	intronic
3	rs79794400	143183239	T	G	4.06	2.38–6.94	2.98E–07	0.040	0.016	0.016	8.01E–04	0	6.50E–04	5.23E–04	0.013	*SLC9A9*	intronic
4	rs118038680	105868416	C	T	3.34	2.14–5.23	1.28E–07	0.063	0.025	0.025	8.74E–04	0	6.50E–04	5.23E–04	0.025	*TET2*	intergenic
4	rs12499648	119953594	A	T	4.33	2.43–7.72	6.66E–07	0.035	0.011	0.011	1.95E–03	1.28E–04	2.13E–03	0.080	5.06E–03	*SYNPO2*	UTR3
7	rs147403806	71797812	T	C	4.49	2.47–8.15	7.93E–07	0.037	0.011	0.012	4.93E–04	1.28E–04	2.13E–03	1.31E–04	0.013	*CALN1*	intronic
9	rs7019089	71107019	A	G	0.56	0.45–0.69	8.12E–08	0.355	0.483	0.481	0.21	0.13	0.29	0.22	0.39	*PGM5*	intronic
9	rs140769784	104088008	T	C	3.50	2.18–5.62	2.10E–07	0.056	0.017	0.018	6.76E–04	1.28E–04	7.22E–05	6.53E–05	0.018	*PLPPR1*	downstream
10	rs11595141	30128609	C	T	4.30	2.55–7.26	4.86E–08	0.049	0.015	0.016	0.09	0.062	0.042	0.090	0.019	*SVIL*	intergenic
15	rs72732628	54124178	C	T	2.13	1.58–2.88	7.04E–07	0.152	0.087	0.088	0.07	0.10	0.047	0.044	0.044	*WDR72*	intergenic
21	rs529360114	46391217	A	G	5.10	2.81–9.24	7.79E–08	0.033	0.012	0.012	2.96E–04	0	7.22E–05	1.31E–04	2.90E–03	*FAM207A*	intronic

† Genes were annotated by using Open Targets (https://www.opentargets.org) and Annover

*Chr* chromosome, *A1* minor allele, *A2* major allele, *OR* odds ratio, *CI* confidence interval, *NA* not assessed

### The expression quantitative trait (eQTL) analysis

We also evaluated the cis-eQTL for the 10 lead variants associated with DG and gene expression data for brain tissue obtained from the GTEx database [25]. We found *RP11-88I18.3* and *TMEM252* gene expressions associated with the rs7019089 variant on *PGM5* locus in the brain cerebellum (Supplementary Table S4). To further identify gene expressions associated with candidate DG risk variants, we also conducted AlphaGenome [26], a recently developed Artificial intelligence tool to explore genomic functions such as eQTLs, and obtained genes associated with candidate DG risk variants. The top 10 data for down- and up-regulated genes are shown in Supplementary Table S5.

### Brain tissue-specific TWAS

To further annotate the candidate causal genes associated with DG, we conducted TWAS using the results of DG-GWAS in 13 brain tissues. In the brain frontal cortex, the *DAPK2* gene on chromosome 15 reached Bonferroni-corrected significance (Z score = 4.13, *P*_Bon_ = 3.68 $\times$10^–5^). The regional association plots for the *DAPK2* locus in DG-GWAS are shown in the supplementary Fig. S1k. The variants in the *DAPK2* locus showed suggestive significance (Top lead variant (rs59606483) showed *P* = 3.66 $\times$10^–5^).

### Genetic correlations across different diseases and traits

We investigated the genetic overlap between our GWAS data and phenotypes from other GWAS by using GWAS summary statistics from the BBJ database [21, 22]. Among the 20 phenotypes analyzed (BMI, brain tumor, cerebral aneurysm, depression, epilepsy, gastric cancer, Hashimoto’s disease, intracerebral hemorrhage, ischemic stroke, myocardial infarction, neuropathic bladder, nephrotic syndrome, osteoporosis, pulse pressure, periodontal disease, systemic lupus erythematosus, type 1 diabetes, type 2 diabetes, and AID disease; Supplementary Fig. S2), we found a significant correlation for pulse pressure (*P* = 0.018).

### *APOE* and *MAPT* locus

**Fig. 3:**
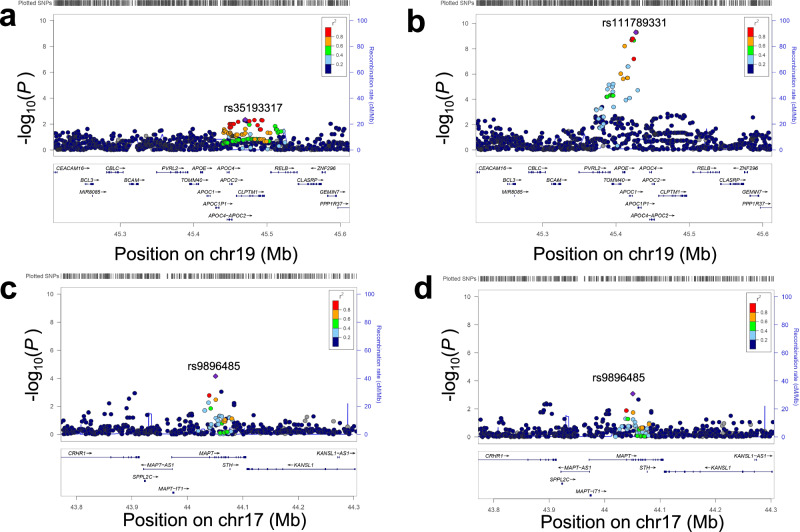
Regional association plot for *APOE* and *MAPT* for DG vs CN and DG vs AD. Regional association plot for (**a**) DG vs CN, **b** DG vs AD in *APOE* on chromosome 19, **c** DG vs CN, and **d** DG vs AD in *MAPT* on chromosome 17

*APOE* has three major allelic characters ε2, ε3, and ε4 [27]. These alleles are determined by two variants (rs7412 and rs429358). The ε4 allele of *APOE* is a strong genetic risk factor for the onset of AD [28]. For DG with *APOE* ε4, the pathology was reported to be a progressive disorder with AD [29]. In contrast, *APOE* ε2 seems to confer a protective effect against AD [30]. For DG, *APOE* ε2 has been demonstrated to increase the risk for the onset [31]. In this context, we assessed the impact of *APOE* alleles on the onset of DG with Japanese subjects. We first investigated the association of DG with the two variants (rs7412 and rs429358) in DG-GWAS and followed by the association between DG and the two variants with AD as controls (Table 2, Fig. 3a, b). The logistic regression analysis of the rs429358 variant for DG with AD as the control group showed a strong association with statistical significance (*P* = 6.$25\times$10^–9^), and the rs7412 variant had no significance (*P* = 0.28). However, DG-GWAS showed no association with rs7412 and rs429358 (*P* = 0.41). The frequencies of *APOE* ε4 alleles in DG, CN, and AD were 6.07, 9.77, and 22.27%, respectively (Table 3). *APOE* ε4 carriers had a higher AD risk than DG (DG-AD; *P*_Fisher_ < 2.2$\,\times$10^–16^, Odd Ratio (OR) = 0.21 (4.84 for AD cases versus DG), and DG versus CN; *P*_Fisher_ = 0.0097, OR = 0.58). We observed that the frequencies of *APOE* ε2 alleles for DG, AD, and CN were 4.67, 2.85, and 4.51, respectively (Table 3). The frequency of *APOE* ε2 carriers for DG showed statistical differences in the frequency of *APOE* ε2 carriers for AD (*P*_Fisher_ = 0.035, OR = 1.70), and no statistical differences for CN carriers (*P*_Fisher_ = 0.72, OR = 1.07).

**Table 2:** Association for APOE and MAPT variants with DG in DG vs CN and DG vs AD

Chr	Pos(hg19)	SNPID	A1	A2	Logistic regression						A1 allele frequency (A1AF)			Gene	Location
					DG vs CN			DG vs AD							
					OR	95% CI	*P*	OR	95% CI	*P*	CN	DG	AD		
17	44050828	rs9896485	C	G	0.65	0.52–0.8	6.99E–05	0.70	0.57–0.86	8.38E–04	0.50	0.40	0.49	*MAPT*	intronic
19	45411941	rs429358	C	T	0.84	0.55–1.28	0.41	0.30	0.20–0.45	6.25E–09	0.1	0.061	0.22	*APOE*	exonic
19	45412079	rs7412	T	C	0.81	0.49–1.34	0.41	1.31	0.80–2.13	0.28	0.05	0.047	0.030	*APOE*	exonic

*Chr* chromosome, *A1* minor allele, *A2* major allele, *OR* odds ratio, *CI* confidence interval

**Table 3:** Frequencies of *APOE* genotypes and alleles in this study

	DG (*n* = 214)		CN (*n* = 12,394)		AD (*n* = 4015)	
	No. of subjects	%	No. of subjects	%	No. of subjects	%
Genotypes						
ε2/2	0	0	30	0	0	0
ε3/2	19	8.88	948	7.67	194	4.83
ε3/3	170	79.44	9115	73.72	2253	56.11
ε4/2	1	0.47	109	0.88	35	0.87
ε4/3	23	10.75	2072	16.76	1313	32.70
ε4/4	1	0.47	120	0.97	220	5.48
Alleles						
ε2	20	4.67	1117	4.51	229	2.85
ε3	382	89.25	21,250	85.73	6013	74.88
ε4	26	6.07	2421	9.77	1788	22.27

AGs are associated with neurofibrillary lesions enriched in 4-repeat (4 R) tauopathy [2]. We considered that the variants in the microtubule-associated protein tau gene (*MAPT*) on chr17p21, which encodes tau proteins, were associated with the risk of DG and also differentiated from AD. Then, we investigated the association for *MAPT* variants in our DG-GWAS and the association between DG and the variants with AD as controls. As shown in regional association plots for *MAPT* locus in the GWAS and in the association analysis with DG using AD as controls (Fig. 3c, d), we did not find the variants to reach suggestive significance (*P* < 1.0$\times$10^–6^). However, we confirmed that the rs9896485 variant in the *MAPT* was nominally associated with DG with protective effects (OR < 0.7) in the DG and in the association analysis with DG using AD as controls (*P* = 6.99$\times$10^–5^ for DG, and *P* = 8.38$\times$10^–4^ with AD as controls, respectively) (Table 2).

## Discussion

We performed DG-GWAS in Japanese subjects for the first time and identified one novel GWS locus on chromosome 10 and nine suggestive loci (Table 1). The lead variant identified as the GWS locus, rs11595141, was closest to the *SVIL* gene. According to eQTL analysis from GTEx database [25], this lead variant is associated with decreased *SVIL* gene expression in the thyroid, although the relation to DG pathogenesis is unknown. *SVIL* encodes supervillin, a large eukaryotic protein from the villin/gelsolin superfamily of actin-binding proteins involved in many cellular processes [32]. There is no direct evidence to date on genes in the locus associated with neurodegenerative disorders. However, the *SVIL* locus was recently identified as a novel hypertrophic cardiomyopathy (HCM) risk locus [33]. Moreover, recent reports have shown that the subjects with HCM demonstrated an increased risk of dementia, mainly AD rather than other dementias [34]. In the genetic correlation analysis using the summary statistics of our DG-GWAS, we found a genetic correlation between pulse pressure and DG. These findings possibly suggest that the neurodegenerative disorder risks, including DG, may be associated with heart diseases, although no information regarding heart disease in DG patients was available in this study.

One of the suggestive loci identified in the GWAS is located downstream of the *PLPPR1* gene. This locus was reported to be associated with the age at diagnosis of Parkinson’s disease (PD), a common neurodegenerative disease with complex clinical features [35]. *PLPPR1* encodes a member of a brain-specific gene family that modulates neuronal plasticity during development, aging, and after brain injury [36, 37]. A previous study reported that *PLPPR1* enhances axonal growth, improves motor behavior, and facilitates functional recovery after neuronal injury using mouse models [36, 37]. The PD-associated locus included missense variants with a predicted destabilizing effect on *PLPPR1*, and the other variants interact with both enhancers and promoters of *PLPPR1* in addition to some other brain-expressed genes [35].

Another suggestive locus identified in the GWAS is located downstream of the *NBAS* gene. The top lead variant at this locus, rs187486988, is unique to East Asian (Table 1). *NBAS* encodes a NBAS subunit of NRZ tethering complex, which is involved in Golgi-to-ER retrograde transport [38]. However, no report of the relation between DG and NBAS was available.

By TWAS using the summary statistics of the GWAS, we identified an association between DG and *DAPK2* in Brain_Frontal_Cortex_BA9. The regional plot for the *DAPK2* locus in the GWAS showed a possible association with DG (Supplementary Fig. S1k). *DAPK2* encodes one of the proteins for the death-associated protein kinase (DAPK) family, consisting of Ser/Thr protein kinases that control various cellular processes [39]. *DAPK2* is involved in apoptosis, autophagy, granulocyte differentiation, and motility regulation [39, 40]. *DAPK1* is a well-known molecule in the DAPK family, and interacts with *Pin1/PINN-1* [41], which regulates dendritic protein synthesis [42] and is implicated in a variety of neurological diseases, including Alzheimer’s disease [43]. *PINN-1* has been shown to regulate neuronal cytoskeleton and Tau protein phosphorylation and modulate neurodegeneration [44, 45]. Functional genetic variants (rs4877365 and rs4878104) of the *DAPK1* gene have been associated with AD and frontotemporal dementia (FTD) [43, 46]. In our DG-GWAS, two functional variants in the *DAPK1* gene showed no association with DG (*P* > 0.1). *DAPK2* is highly homologous to *DAPK1* in the catalytic domains, showing 80% identity at the amino acid level [39, 43]. While the association between *DAPK1* function and neurological disorders is relatively well-known, that of *DAPK2* is poorly understood.

Finally, by focusing on genetic variants of the *APOE* and *MAPT* genes, we examined the differences in the genetic architecture between DG and AD. We did not find any association between *APOE* ε4 carriers and DG risk. Whereas the previous report indicated that the *APOE* ε2 allele was protective for DG risk [31], our study did not replicate this finding. The *MAPT* locus represents two known haplotypes, H1 and H2. H1 haplotype is associated with the risk of AD, CBD, and PSP [11, 12, 47]. The H1 sub-haplotype (consisted from rs1467967, rs242557, rs3785883, rs2471738, rs8070723 and rs7521) [48] was not associated with our GWAS (*P* > 0.05). Our GWAS findings suggested that the *MAPT* H1/H2 alleles are unlikely to be the risk factor for DG. On the other hand, we have identified a novel variant (rs9896485) in the *MAPT* locus associated with a protective effect for DG with possible statistical significance. It is also known that the *MOPB* locus is the common risk locus for CBD and PSP [11, 12], however, there was no association at the GWAS (*P* > 0.1).

However, the present study has some limitations. Statistical power was insufficient to detect the variants with a lower odds ratio (<1.5) in the sample size of our population. Thus, the analyses with additional sample sizes and replication analyses with the other Asian cohorts may provide further insights into the genetic architecture of DG.

Our first GWAS for DG, followed by TWAS and related analysis in the Japanese population, has successfully revealed a novel genetic architecture of DG. We believe that the findings of genetic factors contributing to pathogenesis will provide novel biological and clinical insights and facilitate the medical and pharmaceutical investigations for developing early prediction, preventive measures, and treatment for serious common diseases.

## Supplementary information

Supplementary Table S1

Supplementary Table S2

Supplementary Table S3

Supplementary Table S4

Supplementary Table S5

Supplementary Table S6

Supplementary Figure S1~S3

## Data Availability

The datasets used or analyzed during the current study are available from the corresponding author upon reasonable request.
