# Supplementary material for: Genome and transcriptome-wide association studies identify multiple novel loci for dementia with grain in Japanese
Source: J Hum Genet. 2025 Dec 17;71(5):301–8. doi: 10.1038/s10038-025-01438-7 (PMC13109055; doi:10.1038/s10038-025-01438-7)
Supplement: Supplementary file 7 — Supplementary Figure S1~S3 [file 10038_2025_1438_MOESM7_ESM.pptx]

## Slide 1
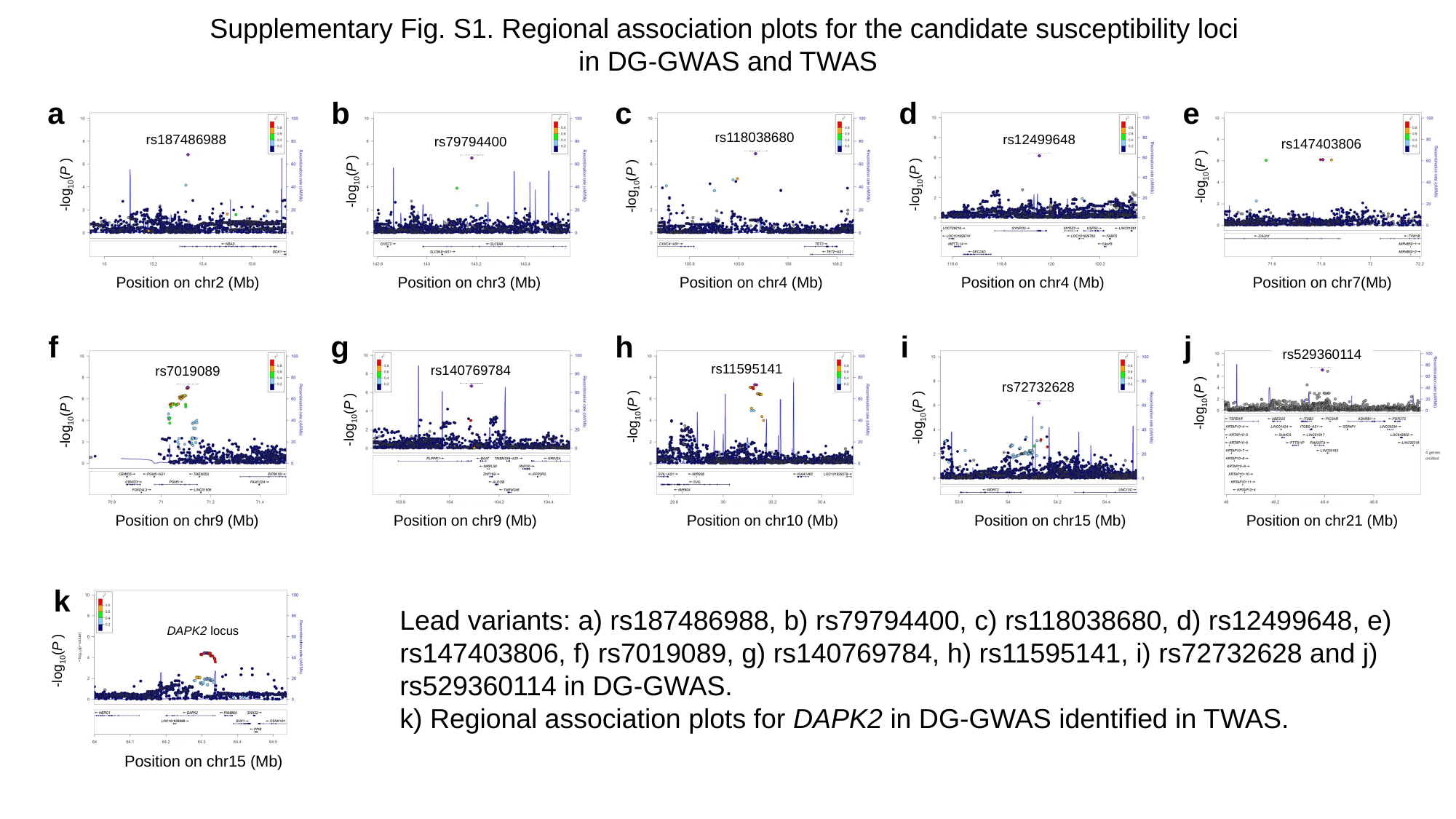

Supplementary Fig. S1. Regional association plots for the candidate susceptibility loci
in DG-GWAS and TWAS
a
b
c
d
e
rs118038680
rs187486988
rs12499648
rs79794400
rs147403806
-log10(P )
-log10(P )
-log10(P )
-log10(P )
-log10(P )
Position on chr2 (Mb)
Position on chr3 (Mb)
Position on chr4 (Mb)
Position on chr4 (Mb)
Position on chr7(Mb)
f
g
h
i
j
rs529360114
rs11595141
rs140769784
rs7019089
rs72732628
-log10(P )
-log10(P )
-log10(P )
-log10(P )
-log10(P )
Position on chr9 (Mb)
Position on chr9 (Mb)
Position on chr10 (Mb)
Position on chr15 (Mb)
Position on chr21 (Mb)
k
Lead variants: a) rs187486988, b) rs79794400, c) rs118038680, d) rs12499648, e) rs147403806, f) rs7019089, g) rs140769784, h) rs11595141, i) rs72732628 and j) rs529360114 in DG-GWAS.
k) Regional association plots for DAPK2 in DG-GWAS identified in TWAS.
DAPK2 locus
-log10(P )
Position on chr15 (Mb)

## Slide 2
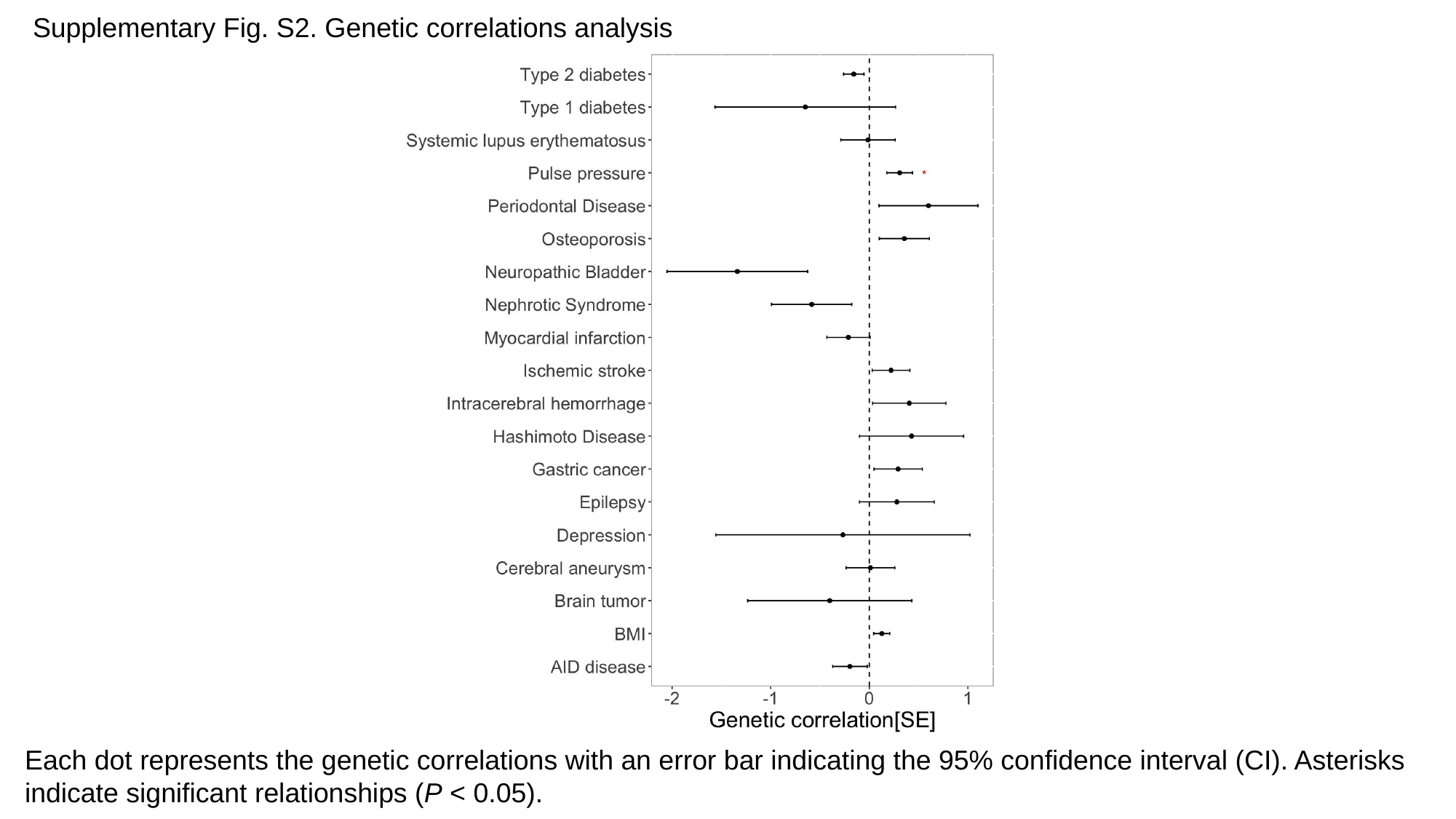

Supplementary Fig. S2. Genetic correlations analysis
Each dot represents the genetic correlations with an error bar indicating the 95% confidence interval (CI). Asterisks indicate significant relationships (P < 0.05).

## Slide 3
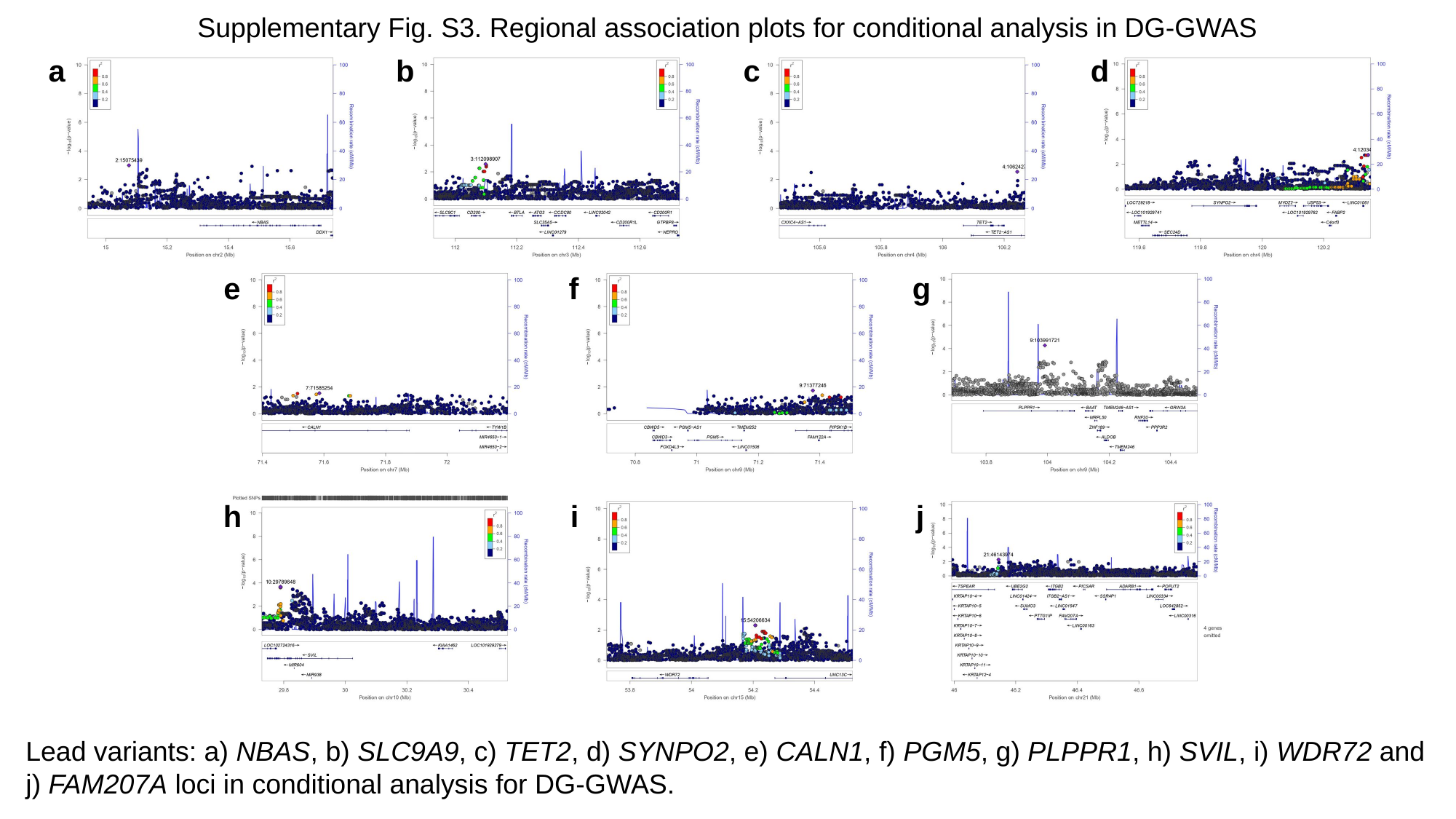

Supplementary Fig. S3. Regional association plots for conditional analysis in DG-GWAS
a
b
c
d
e
f
g
h
i
j
Lead variants: a) NBAS, b) SLC9A9, c) TET2, d) SYNPO2, e) CALN1, f) PGM5, g) PLPPR1, h) SVIL, i) WDR72 and j) FAM207A loci in conditional analysis for DG-GWAS.
